# The Impact of Steep Trendelenburg Position on Intraocular Pressure

**DOI:** 10.3390/jcm11102844

**Published:** 2022-05-18

**Authors:** Matteo Ripa, Chiara Schipa, Nikolaos Kopsacheilis, Mikes Nomikarios, Gerardo Perrotta, Carlo De Rosa, Paola Aceto, Liliana Sollazzi, Pasquale De Rosa, Lorenzo Motta

**Affiliations:** 1 Ophthalmology Unit, Fondazione Policlinico Universitario A. Gemelli IRCCS, 00168 Rome, Italy; matteof12@gmail.com; 2 Catholic University “Sacro Cuore”, 00135 Rome, Italy; paola.aceto@policlinicogemelli.it (P.A.); liliana.sollazzi@unicatt.it (L.S.); 3 Department of Emergency, Anesthesiological and Reanimation Sciences, Fondazione Policlinico Universitario A. Gemelli IRCCS, 00168 Rome, Italy; 4 East Kent Hospitals University NHS Foundation Trust, Kent and Canterbury Hospital Ethelbert Road, Canterbury CT1 3NG, UK; n.kopsacheilis@nhs.net; 5 New Hayesbank Ophthalmology Services, Cemetery Lane, Kennington, Ashford TN24 9JZ, UK; 6 Department of Ophthalmology, William Harvey Hospital, East Kent Hospitals University NHS Foundation Trust, Ashford TN24 0LZ, UK; mikes.nomikarios@nhs.net (M.N.); lorenzo.motta@nhs.net (L.M.); 7 GI Surgery Department, University College London Hospitals NHS Foundation Trust, London NW1 2PG, UK; gerardo.perrotta@nhs.net; 8 Department of Ophthalmology, A. Cardarelli Hospital, 80131 Naples, Italy; derosacarlo96@gmail.com (C.D.R.); pasquale_de_rosa@hotmail.it (P.D.R.)

**Keywords:** intraocular pressure, steep Trendelenburg position, laparoscopic surgery, robotic surgery, ophthalmology

## Abstract

Intraocular pressure occurring during the Trendelenburg position may be a risk for postoperative visual loss and other ocular complications. Intraocular pressure (IOP) higher than 21 mmHg poses a risk for ocular impairment causing several conditions such as glaucoma, detached retina, and postoperative vision loss. Many factors might play a role in IOP increase, like peak expiratory pressure (PIP), mean arterial blood pressure (MAP), end-tidal CO_2_ (ETCO_2_) and surgical duration and some others (anaesthetic and neuromuscular blockade depth) contribute by reducing IOP during procedures requiring both pneumoperitoneum and steep Trendelenburg position (25–45° head-down tilt). Despite transient visual field loss after surgery, no signs of ischemia or changes to the retinal nerve fibre layer (RNFL) have been shown after surgery. Over the years, several studies have been conducted to control and prevent IOPs intraoperative increase. Multiple strategies have been proposed by different authors over the years to reduce IOP during laparoscopic procedures, especially those involving steep Trendelenburg positions such as robot-assisted laparoscopic prostatectomy (RALP), and abdominal and pelvic procedures. These strategies included both positional and pharmacological strategies.

## 1. Introduction

Intraocular pressure (IOP) is defined as “the fluid pressure inside the eye”. As pressure is a measure of force per area, IOP measurement involves the magnitude of the force exerted by the aqueous humour on the internal surface area of the anterior eye [1]. According to the American Academy of Ophthalmology [2], normal IOP is 10 mm of mercury (mmHg) to 21 mmHg. IOPs higher than 21 mmHg pose a risk for ocular impairment causing several conditions such as glaucoma, detached retina, and postoperative vision loss [3,4,5].

Many surgical procedures require a specific body positioning in which the patient must be placed in a steep Trendelenburg position (STP) (25–45° head-down tilt), such as robot-assisted laparoscopic prostatectomy (RALP) [6] and those used in colorectal [7] and gynaecological surgery [8]. This position uses gravity to pull the abdominal viscera away from the operative field but is non-physiological and may have significant negative physiological effects when maintained for long periods of time [6].

Furthermore, when compared to laparoscopic and open pelvic surgery, STP in robot-assisted pelvic surgery (RAPS) has fewer postoperative complications. STP is associated with a significant decrease in the risk of venous thromboembolism because it improves venous return from the lower limbs and lowers blood stasis. Although it increases cardiac preload by increasing central venous pressure from 80 to 305% [9], Katayama et al. found no difference in cardiac complications in surgical procedures performed in STP compared to laparoscopic/open pelvic surgery, suggesting that STP has little impact on postoperative cardiac complications [10].

Despite giving many surgical advantages (better organ visualization and access), extreme changes in patients’ posture while undergoing surgery can cause a rise in IOP leading to changes that can dramatically affect clinical outcomes [11]. Indeed, at pressures exceeding the ocular perfusion pressure (OPP), raised IOP causes compression of the vasculature, resulting in retinal ischemia and potential vision loss.

Blood pressure higher than 20 mmHg for 5 min determines a reduction of retinal, choroidal, and optic nerve blood flow [12], thus interfering with the delivery of essential neurotrophins from the brain to the retina, as previously demonstrated in rats [13].

## 2. Physiologic Changes Related to Trendelenburg Position

IOP is regulated by aqueous humour production, aqueous humour drainage, auto-regulation and control of choroidal blood volume, vitreous humour volume and extraocular muscle tone [14]. While the production of aqueous humour is stable, the outflow of aqueous humour to the venous system may be affected by choroidal blood volume, vitreous humour volume and extraocular muscle tone.

Many surgical procedures require pneumoperitoneum, where the air is insufflated into the abdominal cavity, and subsequent Trendelenburg position (TP), where the patient is positioned supine on the table with the head tilted below the feet at an angle of roughly 16°, and up to 25–40° in STP: both can cause hemodynamic alterations that may influence IOP increasing venous congestion [15].

After induction of anaesthesia, there is an initial IOP decrease from baseline (IOP is reduced significantly more by propofol compared to volatile anaesthetics [16,17]) followed by a slight increase after insufflation of pneumoperitoneum [16,17,18,19].

Afterwards, the venous congestion increases both central venous pressure (CVP) and IOP. The latter increases in a time-dependent manner: mean IOP doubling within 60 min and, in 25% of cases, tripling within 120 min. This IOP increase may be exacerbated by the pneumoperitoneum-induced increase in partial pressure of carbon dioxide (PaCO_2_) [20]. Indeed, the carbon dioxide (CO_2_) insufflation leads to an increase in IOP due to decreased venous return and increased episcleral venous pressure. An increase in venous pressure, which is then in turn transmitted to the episcleral veins and from these to the capillaries and arterioles, also contributes to decreased optic nerve and ocular perfusion. The choroidal expansion may also lead to elevation [21]. This increased IOP then leads to decreased ocular perfusion pressure (OPP).

IOP, however, normally plateaus after about 30–60 min and decreases after return to the supine position [18].

## 3. Factors Determining Increased IOP in STP

Many authors investigated the role of several intraoperative factors which could lead to an IOP increase in surgical procedures requiring STP. In 2009 Awad et al. [22] and in 2017 Blecha et al. [21] stated that peak expiratory pressure (PIP), mean arterial blood pressure (MAP), end-tidal CO_2_ (ETCO_2_) and duration of procedure were significant predictors of IOP. ETCO_2_ led to choroidal vasodilation increasing IOP. Indeed, the continued absorption of intraperitoneal CO_2_ and/or increased pressure on the diaphragm, resulting in lower delivered tidal volumes, increased ETCO_2_ causing the increase of arterial pCO_2_ levels, leading to vasodilation in the choroid plexus and an increase in IOP.

PIP might increase IOP during STP because an increase in intrathoracic pressure leads to increases in CVP which may reduce the aqueous humour outflow through the episcleral veins [22].

MAP and IOP are likely related, as increases in mean blood pressure led to increases in aqueous humour ultrafiltration by means of increased ciliary artery pressure, and thus an increase in IOP [23,24].

A few years later, Molloy et al. [25] and Adisa et al. [18] investigated the role of the duration of surgery in predicting increased IOP, stating that prolonged duration of surgery was an important factor in attaining more dangerous elevations of IOP (Table 1).

## 4. Factors Reducing IOP in STP

In 2015 Yoo et al. [26] demonstrated the role of both anaesthesia and depth of neuromuscular blockade (NMB).

He observed that propofol causes attenuation of IOP when compared to sevoflurane inhalational anaesthesia in STP patients. However, Gofman et al. [27] and Sator-Katzenschlager et al. [17] showed no change in intraocular pressure in supine patients undergoing propofol or sevoflurane.

Moreover, NMB plays a role in IOP decrease because of the greater relaxation of extraocular muscles which facilitates aqueous humour drainage.

In 2015, Raz et al. [28] investigated a modification of head position while in STP. The modified Z position consisted of placing the patient in STP and then positioning the head and shoulders horizontally. This change in position showed a decrease in IOP when compared to patients in standard STP positions.

## 5. Timing

Using an angle of 30°, Molloy et al. [25] and Mondzelewski et al. [29] found that IOP rises and reaches a relative plateau between 30 and 60 minutes as the patient is maintained in STP.

In 2017 Ozcan et al. [30] analysed the effect of STP on IOP during robotic surgery including 43 RALP cases, finding that the highest IOP was at the end of ST under pneumoperitoneum, whereas in 2020 Shirono et al. [31] discovered that IOP increased 1 h after induction of pneumoperitoneum in STP, remaining elevated in a time-dependent manner during STP.

Although several studies report that STP increases IOP, few studies investigated the pattern of IOP elevation during STP and in 2021 Kondo et al. [32] conducted a study to quantify pressure changes over time in patients assuming the steep Trendelenburg position during robotic-assisted laparoscopic prostatectomy in order to clarify the pattern of IOP elevation during surgery.

They measured IOP: before induction of anaesthesia in the supine position (T0); 30 (T1), 90 (T2), and 150 min after assuming the Trendelenburg position (T3); and 30 min after returning to the supine position (T4). Their results showed that IOP increased approximately by 5 mmHg 30 min after assuming the STP, reaching the plateau after 90 min, and returning at the baseline level 30 min after returning to the supine position.

## 6. Magnitude of Mean Change in IOP

Several investigations have been carried out to examine IOP changes at several discrete time points during different surgical procedures involving STP.

As IOP changes primarily during abdominal insufflation in supine position and TP, the majority of measurements were taken at specific time intervals such as baseline, after abdominal insufflation, after TP initiation, after 60 min of TP, and so on. Many of these investigations also examined whether IOP fluctuations differed depending on the surgical position, the anaesthetic medicines used, the depth of neuromuscular blockade, and specific factors including PIP, ETCO_2_, and MAP. However, none of them included individuals with a high IOP, such as those with ocular hypertension or glaucoma, or patients with an IOP of more than 30 mmHg. Therefore, the mean baseline IOP was always less than 21 mmHg [28,33,34,35,36,37].

According to a systematic review published in 2019 by Van Wicklin, IOP increases significantly after abdominal insufflation in the supine position (+3.5 mmHg, *p* < 0.05), when the patient is positioned in TP (+4.4 mmHg, *p* < 0.001), and with extended time in the TP (+2.6 mmHg, *p* = 0.001), reaching values as high as 35 mmHg after 180 to 240 min in the TP. Furthermore, mean IOP also tends to drop significantly during induction of anaesthesia (−5.2 mmHg, *p* = 0.001) and before arousal from anaesthesia (−7.5 mmHg, *p* = 0.001) [38].

Regarding the specific anaesthetic used during the surgical procedure, Kim et al. reported that administering dexmedetomidine in addition to inhaled anaesthetics reduced average IOP by 6 mmHg after 1 h in STP compared to administering inhaled anaesthetics only [39].

Furthermore, IOP tends to decrease with posture. Mondelwesky et al. compared the changes in IOP in a robot-assisted laparoscopy group undergoing STP with a control group undergoing open surgery in the horizontal position (group 2) and laparoscopic cases in the horizontal position (group 3). Although baseline IOP was similar, IOP plateaued at 29.9 mmHg (95% confidence interval, 27.4–32.5), 19.9 mmHg (95% confidence interval, 17.6–22.3), and 22.8 mmHg (95% confidence interval, 20.2–25.4) for groups 1, 2, and 3, respectively, from 60 min until the conclusion of the case. In comparison to the open and the laparoscopic controls, IOPs in Group 1 achieved a plateau at a level greater than twice the initial IOP, indicating a considerable rise in IOP during STP [29].

## 7. Pathogenesis of Postoperative Vision Loss

Although postoperative visual loss (PVL) incidence after non-ocular surgery has been estimated to be as low as 0.0002% [40,41], the incidence of postoperative vision loss specific to patients undergoing surgery in the steep Trendelenburg position remains unknown.

Higher IOP may injure the optic nerve because of blood flow reduction determining ischemic optic neuropathy (ION), which is the most common cause of postoperative visual loss (PVL) [41,42]. Hence, ION can be the result of decreased blood supply from the arteries of the optic nerve or can occur due to decreased venous outflow [25,42,43].

Several studies report that increased IOP associated with the Trendelenburg position poses a greater risk for postoperative vision loss in patients who have existing ocular disease compared to patients without ocular disease [19,20,26,29,34,44].

Patients with existing ocular conditions, such as elevated baseline IOP, glaucoma and ocular hypertension, are more likely to develop postoperative vision loss compared to those without any ocular disease [15,29,34,42]. Moreover, both elderly patients [15,29,42] and cardiopathic patients [19] may be likely to develop postoperative visual loss compared to younger and non-cardiopathic patients, respectively.

Undoubtedly, increases in IOP and subsequent risk for postoperative vision loss are strictly related to the amount of time the patient stays in the Trendelenburg position [21,24,25,34].

## 8. Postoperative Visual Defects

Steeper degrees of Trendelenburg increase the likelihood of postoperative visual defects because they put the patient’s body under more physiologic stress leading to several postoperative complications such as ischemic optic neuropathy, visual acuity, optic nerve changes, and visual field defects.

### 8.1. Ischemic Optic Neuropathy

An elevated IOP decreases the perfusion pressure to the optic nerve, leading to increased risks of ION and visual loss, which significantly affect quality of life.

Although ION after prostate surgery is uncommon, Foroozan described a 61-year-old man who developed shock-induced anterior ischaemic optic neuropathy (SIAION) after a radical prostatectomy in 2004. Visual loss occurred three days after surgery [45].

Subsequently, Weber et al. first reported a case of a 62-year-old patient who had posterior ischemic optic neuropathy after a robot-assisted procedure lasting 6 h 35 min and with a blood loss of 1200 mL. The patient complained of “purple vision” along with loss of the inferior visual fields in both eyes on the first postoperative day. Automated Humphrey visual fields revealed bilateral inferior altitudinal defects, which were stable three months later during follow-up, associated with the pallor of the supertemporal optic disc on both sides. In the second case, a 64-year-old man underwent a laparoscopic prostatectomy and subsequently complained of “dark eyesight”. His vision had light perception in the right eye and no light perception in the left eye, both of which were accompanied by temporal pallor of the optic disc. The optic disc pallor persisted for two months following surgery, and the patient’s vision did not improve [46].

The first case of ION rising during laparoscopic proctocolectomy was described by Mizrahi et al. in 2011. The operation lasted over 6 h and the patient complained of cloudy vision on the first postoperative day. His visual acuity was light perception in his right eye and counting fingers in his left. Despite some improvement in visual acuity during follow-up visits, a superior altitudinal field defect was detected [47].

Nevertheless, Hoshikawa et al. [34] determined that the increase in IOP occurred in patients undergoing robot-assisted prostatectomies in STP could not lead to ischemia or postoperative vision loss as best-corrected visual acuity (BCVA) and the retinal nerve fibre layer (RNFL) showed no changes after the procedure.

Although the aetiology of ION is unclear, optic nerve injury is most likely caused by one of two pathophysiological mechanisms: decreased blood supply (ischaemia) from the optic nerve’s arteries or venous stasis because of reduced venous outflow. As a result, the pathogenesis of ION after surgery could be associated with severe arterial hypotension or significant blood loss during general anaesthesia, haemodilution from intravenous fluid over infusion, or elevated ocular pressure.

When ION risk factors such as age and smoking are present, both conditions may result in ischaemic optic nerve injury with severe permanent visual impairment [48].

### 8.2. Visual Fields and Optic Nerve Changes

To investigate the effect of IOP rise on the visual field, RNFL thickness, and optic disc morphology, in 2015 Taketani et al. [20] reported transient but significant symptomless unilateral visual field defects one week after surgery in 28% of the patients who underwent RALP despite no abnormal findings in the fundus, RNFL thickness or optic disc morphology. All pathological findings returned to normal within 3 months after the surgery, suggesting ION or other ischemic factors as a possible mechanism.

Furthermore, an increase in IOP during STP may have a detrimental impact on intracranial pressure (ICP), resulting in larger optic nerve sheet diameters (ONSD).

In 20 patients undergoing RALP, Kim et al. [49] found that the ONSD increased by 12.5% (0.6 mm) during CO_2_ pneumoperitoneum and STP. Following that, Chin et al. [50] found a 12.5% (0.6 mm) increase in ONSD in 20 patients undergoing RALP after CO_2_ insufflation and STP. Similarly, Balkan et al. reported a 0.34 mm rise in ONSD after CO_2_ insufflation and a 0.34 mm increase in STP during RALP compared to baseline [51].

Recently, Kim et al. investigated the effects of sevoflurane and propofol in patients undergoing Robot-Assisted Laparoscopic Gynaecological Surgery. 40 min after induction of pneumoperitoneum in TP, ONSD increased significantly in the sevoflurane group compared to the propofol group. As a result, in patients undergoing robot-assisted surgery, anaesthesia with propofol produced lower increases in ONSD compared to anaesthesia with sevoflurane [52].

The rise in ICP generated by CO_2_ pneumoperitoneum and the TP is related to ONSD. The head-down position, as well as head rotation and/or flexion, increase ICP due to a fluid shift and venous engorgement, as well as a blockage of cerebral venous outflow caused by the increased central venous pressure [53].

Nevertheless, Awad et al. [54] reported that 56 patients without any ocular disease who underwent robotic hysterectomies and 24 patients who underwent RP did not develop any visual impairment after the procedures. Indeed, visual acuity, RNFL thickness and ganglion cell complex (GCC) thickness were normal and did not differ significantly preoperatively and 3 months postoperatively.

Mizumoto et al. confirmed the previous findings, stating that 44 eyes of 22 patients undergoing RALP had no ocular complications. Indeed, mean deviation, pattern standard deviation (measured by the Humphrey field analyser) and the mean visual acuity, showed no statistically significant difference before and after surgery. Furthermore, the thickness of the GCC and RNFL measured at each location, as well as the thicknesses of the central fovea measured before and after surgery, did not differ significantly [55]. In 2021, Goel et al. found no deterioration in visual acuity (measured 6 h after surgery) in 100 patients undergoing robotic surgery in STP [56].

Afterwards, Wen et al. [57] reported that rates of ocular complications in non-robotic radical prostatectomy (RP) are comparable with those undergoing RALP, stating that visual complications following RP were related to either prolonged surgical time or excessive blood loss. In 2017 Nishikawa et al. [36] demonstrated that RALP with 25° Trendelenburg position reduced the risk of ophthalmologic complications without prolonging operative time and/or increasing blood loss during surgery compared with 30° Trendelenburg position.

## 9. Preventive Strategies for Rising Intraocular Pressure

Multiple strategies have been proposed by different authors over the years to reduce IOP during laparoscopic procedures, especially those involving steep Trendelenburg positions such as RALP, abdominal, and pelvic procedures.

These strategies included both positional [24,25,28] and pharmacological strategies [37,58,59,60,61,62].

### 9.1. Positional Strategies

Although STP provides many surgical advantages, it may increase the risk of several ocular complications [19,20,43,44].

In 2012 Ghomi et al. [35] completed all their cases successfully using 16° Trendelenburg without the need to modify the table tilt in robot-assisted gynaecologic surgery. There were no perioperative complications, and the operating times were similar to those in previous reports. While in standard STP the patient is supine and has a head-down tilt of a maximum of 45°, Raz et al. [28] proposed the modified Z Trendelenburg position to maintain the head and shoulders in a horizontal position while tilting the patient in a head-down ST position. This position decreased IOP without compromising the operative field, anaesthesia, or the procedure.

Implementing periodic intraoperative position changes or rest periods in the supine position (or positions where the ocular level is above the heart) can help reduce IOP. Molloy and Watson [58] implemented a Level Supine Intervention (LSI), consisting of periodic rest periods in the supine position during urological, colorectal and gynaecological laparoscopic procedures in STP, and found that it significantly decreased mean IOP; however, these positional changes increase the operative time, as they involve undocking and redocking of the robot and repositioning of the patient [43] (Table 2).

### 9.2. Pharmacological Strategies

The choice of anaesthetic agent might also play a role, as Totally Intravenous Anaesthesia (TIVA) with propofol has been shown to mitigate the rise in IOP during lower abdominal laparoscopic procedures in the Trendelenburg position, when compared to inhalational anaesthesia with sevoflurane [37]. This confirmed the findings of Agrawal et al. [59] regarding the induction and maintenance of anaesthesia with propofol which was found to be the most effective option for mitigating the increase in IOP in adult patients undergoing surgery in the Trendelenburg position.

Pharmacological strategies have also been advocated: a randomised double-blinded placebo-controlled trial by Kitamura et al. [60] found a significant reduction in IOP when administering continuous IV dexmedetomidine to patients undergoing RALP who were anesthetised with propofol, while Molloy et al. found the intraoperative administration of dorzolamide-timolol eyedrops (a carbonic anhydrase II inhibitor, and timolol, a topical beta-adrenergic receptor blocker, which decrease IOP by decreasing the production of aqueous humour) to be effective both at reducing elevated IOP (>40 mmHg) [58] and at maintaining a lower IOP when used prophylactically [61] during RALP and gynaecological procedures. A case report by Lee et al. also described the successful reduction in IOP in a patient with glaucoma undergoing RALP with IV acetazolamide followed by a slow infusion of 20% mannitol [33]. While intraoperative treatment of raised IOP (>35–40 mmHg) could be warranted, there is currently little evidence of the correlation between increased IOP and retinal changes, therefore the practice of prophylactically decreasing IOP with pharmacological interventions in patients with healthy ocular systems remains questionable.

In 2018 Mathew et al. [62] conducted a randomised controlled masked interventional trial of patients undergoing RALP treated with either artificial tears or brimonidine tartrate 0.2% preoperatively. However, a preoperative loading of brimonidine tartrate 0.2% failed to show a difference in IOP when compared to a placebo (Table 3).

## 10. Implications for Practice

Should patients undergoing surgery in STP receive a preoperative ophthalmologic examination?

Preoperative ophthalmologic examinations may be helpful in identifying patients at risk for postoperative vision loss or other ocular complications as a rise in IOP may be more likely to harm elderly patients and patients who are predisposed to developing glaucoma [20,44].

In 2012 Molloy [63] developed an observation scale that correlated with statistically significant changes in intraoperative IOP. The scale was named the Molloy/Bridgeport Anaesthesia Observation Scale (MBOS) and was used to assess the need for treatment interventions to prevent a rise in IOP levels. The scale consisted of the presence or absence of 3 critical areas of observation: (1) eyelid oedema, (2) corneal/conjunctival oedema (chemosis), and (3) ecchymosis. Since measuring IOP requires both instruments and experienced personnel, the MBOS could assist the anaesthesia team in determining if and when the IOP may increase.

In 2018 Grosso et al. [64] recommended a risk assessment to help identify individuals who may be at risk for ocular complications following prolonged laparoscopic or robot-assisted laparoscopic procedures. Patients with a diagnosis of glaucoma or those who are currently using glaucoma medications, individuals with a history of ocular trauma or surgery, and individuals undergoing minimally invasive or robot-assisted laparoscopic surgery anticipated to last longer than 1 h, should have IOP monitored intraoperatively.

In 2019 Aceto et al. [65] suggested using STP only for the time strictly necessary for surgery to be performed, using a position tailored to the pelvic operatory field of the subject and avoiding extreme TP (>30°) in high-risk patients.

Despite the increase in IOP during STP, most patients tend to tolerate it well; unfortunately, there is a small risk of postoperative blindness. As surgical time and ETCO_2_ are strong predictors of IOP increase in STP, they should be carefully monitored.

In recent years, many authors have investigated the role of ONSD increases in identifying how increased IOP in TP affects ICP. Many authors examined the role of Ultrasonographic measurement of ONSD in identifying ICP changes during STP, confirming that ONSD increases in TP with pneumoperitoneum, as STP and pneumoperitoneum have a detrimental effect on cerebral hemodynamic physiology [49,51,52,53].

Kumari et al. suggested in 2021 [66] that preoperative ocular examination and regular monitoring of ONSD as a representative of increased ICT and IOP during the intraoperative period should be performed to ensure early awareness of the surgical team, implement early interventions to reduce ICT and IOP as needed, and thus reduce intraoperative ocular complications and postoperative vision loss.

## 11. Conclusions

Although IOP increases might lead to visual loss, few studies report ocular function impairment during surgical procedures in STP.

Over the years, several strategies have been developed to reduce and prevent IOP increases in STP, such as administering specific drugs [37,58,59,60,61,62] or altering TP [25,28,35]. Despite the absence of specific guidelines, patients at risk of developing glaucoma or other ocular complication may benefit from preoperative risk assessments and intraoperative IOP monitoring.

Further research regarding the magnitude of increases in IOP in patients undergoing Trendelenburg is required, especially in those with glaucoma and ocular hypertension. To provide comparable data, future studies should use standard time points for measurement. Additionally, researchers should include patients with glaucoma, ocular hypertension, or any other eye diseases in order to establish whether certain variables influence the strength of the association between STP and IOP.

## Figures and Tables

**Table 1:** Factors determining increased IOP in STP.

Author—Year	Factors	Surgical Procedure/Angle Used
Awad et al. [22]—2009	PIP	RALP (25°)
	ETCO_2_	
	MAP	
	Duration of procedure (min)	
Molloy et al. [25]—2011	Duration of procedure (min)	Laparoscopic surgery (30°)
Adisa et al. [18]—2016	MAP	Laparoscopic surgery (30°)
Blecha et al. [21]—2017	MAP	RALP
	PIP	(45°)

Abbreviations: STP (steep Trendelenburg position); IOP (intraocular pressure); peak expiratory pressure (PIP), mean arterial blood pressure (MAP), end-tidal CO_2_ (ETCO_2_); minutes (min); robot-assisted laparoscopic prostatectomy (RALP).

**Table 2:** Positional strategies to prevent increased IOP in STP.

Author—Year	Type of Strategy	Explanation
Ghomi et al. [35]—2012	TP reduction	Using 16° TP for RAGS
Raz et al. [28]—2015	Modified Z Trendelenburg position	Patients’ head and shoulder positioned at the same level in RALP
Molloy et al. [58]—2016	LSI	Periodic rest periods in supine position for urological, colorectal and gynaecological laparoscopic procedures

Abbreviations: STP (steep Trendelenburg position); TP (Trendelenburg position); IOP (intraocular pressure); robot-assisted laparoscopic prostatectomy (RALP); robotic-assisted gynaecologic surgery (RAGS); Level Supine Intervention (LSI).

**Table 3:** Pharmacological strategies determining IOP reduction in STP.

Author—Year	Drug Administered	Results
Agrawal et al. [59]—2013	Propofol/thiopentone for induction and propofol/1% isoflurane for maintenance	Induction and maintenance with propofol TIVA is the best option as induction with propofol decreased IOP by almost 50%
Molloy et al. [61]—2014	Dorzolamide-timolol when IOP exceeded 40 mmHg	IOP reduction
Molloy et al. [58]—2016	Dorzolamide-timolol after induction of anaesthesia and when IOP exceeded 40 mmHg	IOP reduction
Kaur et al. [37]—2018	Anaesthesia using intravenous propofol/sevoflurane	IOP is significantly greater (*p* < 0.01) in patients treated with sevoflurane compared with those treated with propofol.
Kitamura et al. [60]—2018	Dexmetomidine	Dexmedetomidine combined with propofol decreases IOP in the steep Trendelenburg position during RALP
Mathew et al. [62]—2018	Brimonidine tartrate 1% preoperatively	No significant differences with placebo

Abbreviations: IOP (intraocular pressure); robot-assisted laparoscopic prostatectomy (RALP); Totally Intravenous Anaesthesia (TIVA).

## Data Availability

Not applicable.
